# A starfish-inspired 4D self-healing morphing structure

**DOI:** 10.1038/s41598-024-71919-w

**Published:** 2024-09-25

**Authors:** Raman, Susanna Labisch, Jan-Henning Dirks

**Affiliations:** Biomimetics-Innovation-Centre, Hochschule Bremen – City University of Applied Sciences, Bremen, Germany

**Keywords:** Bioinspired materials, Actuators

## Abstract

Inspired by the starfish's unique ability to achieve flexibility and posture-holding with minimal energy expenditure, we present a novel bioinspired morphing structure. Our two-component design, consisting of a thermoplastic mesh and elastomeric jacket, effectively mimics the functions of the starfish's ossicles, mutable collagenous tissues, and derma. This structure exhibits a remarkable combination of self-healing, time-dependent shape memory, and self-posture-holding properties. Systematic variations in mesh geometry demonstrate precise control over structural stiffness and thermal response, enabling customization for specific applications. The structure's scalability and ease of fabrication further enhance its adaptability. We experimentally demonstrate the potential of our biomimetic morphing structure using several prototypes. This work lays the foundation for developing a new type of versatile morphing structures with applications in diverse fields, including robotics, biomedical devices, and adaptive structures.

## Introduction

Morphing structures are load-bearing structures designed to alter their shape, allowing them to adapt to different operational requirements or external conditions^1,2^. These structures are increasingly gaining attention in research and application across various sectors, including automotive, aerospace, and power plants^3–7^. Traditionally, standard mechanical joints are used to design such morphing structures, where rigid separate elements are connected to kinematic chains using joints with low friction and predetermined degrees of freedom. Another approach involves using complaint monolithic mechanisms, where flexible elements are used to create a structure and through their deflections, the structure can adapt to new shapes^8,9^.

The concept of 4D structures adds an exciting dimension to this field. In 4D structures, the transformation is triggered by stimuli over time, such as temperature, humidity, or other environmental changes^10,11^. Recent advancements have significantly extended the capabilities of 4D structures. For instance, voxelated machines have emerged as a novel approach, leveraging the precise control of material properties at the voxel level to create complex, adaptable structures with high degrees of functionality^12^. Similarly, bio-inspired micro-origami 4D tessellations have been developed, offering new ways to achieve self-morphing behaviors through intricate geometric designs^13^. The integration of liquid–metal-network actuators into an elastomer matrix combines the flexibility and responsiveness of liquid metals with the durability of polymeric networks, enabling the creation of structures that are both highly adaptable and responsive to external stimuli, capable of changing shape or function as needed^14^. Additional studies have further expanded the capabilities of 4D printing. For example, research on adaptive metamaterials by functionally graded 4D printing has demonstrated how gradient-based material properties can be programmed during fabrication to create self-folding or self-coiling structures that respond to environmental changes^15^. Similarly, geometric design pattern-driven single-material 4D printing for self-morphing actuators has shown that even single-material systems can achieve sophisticated morphing behaviors when designed with appropriate patterns^16^.

These morphing structures, while effective in achieving desired shape changes, share a common limitation: they require continuous energy input and active control to maintain their posture. Conventional frictionless mechanical joints rely on external control systems to hold the structure in place; without this, the structure may revert to its original shape or become unstable. Similarly, compliant mechanisms, which depend on the deflection of flexible elements, need constant external forces to maintain their shape. Even in 4D structures, where shape change is triggered by environmental stimuli, an ongoing external force is often required to sustain the new configuration. To minimize the requirement for a continuous energy supply to uphold a structure's geometry, locking mechanisms are occasionally utilized to secure these structures in the desired configuration^17–19^. However, these structures are not able to hold their changed morphological position by themselves. Very few morphing structures are designed in such a way that they have more than one stable state and can hold their shape without requiring any external energy input^20,21^. However, still energy and control are essential for maintaining alternative morphological positions.

In the last years a few bio-inspired solutions for the mechanical challenges of morphing structures have been suggested, mostly focusing on morphogenesis and actuation^22–24^. We here present a new bio-inspired approach to address the energy consumption issue faced by morphing structures. Our bio-inspired design is based on the starfish skeleton, an echinoderm belonging to class Asteroidea. Starfish can bend, move and twist their arms with great degrees of freedom^25,26^. Alongside its structural versatility, the starfish skeleton has the unique capability to be able to hold any body posture with minimal expenditure of energy^27,28^.

Simply stated, starfish skeletons consist of thousands of small bone-like magnesium calcite structures called ossicles^29,30^. These ossicles are connected by small inter ossicular muscles (IOMs) and form a complex mesh with ridge-shaped geometrical patterns^31,32^. This whole assembly of ossicle-IOMs is connected and covered with mutable collagenous tissues^25,31^. Using neural control mechanisms, starfish can control the stiffness of this collagenous tissue to hold their posture for prolonged periods^27,28^. These unique capabilities make starfish an excellent example for a bio-inspired energy-efficient morphing structure in nature.

To better understand the functional principles and detailed morphology of the complex starfish skeleton, we initially performed high-resolution X-ray CT imaging on *Asterias rubens* specimens. Based on our findings we then assigned specific technical roles to different skeleton components, depending upon their functions during morphing and posture-holding processes. Subsequently, we identified commercially available and easy to process materials capable of fulfilling these roles and designed a morphing structure inspired by the starfish skeleton. The resulting morphing structure exhibits the capability to maintain any posture without the need for external energy or control. Alongside, it exhibits a time-dependent shape memory effect (4D) which allows to design smart morphing structures. Our principle also allows for full customizability of mechanical and thermal properties, scalability in length, width, and depth, and shows self-healing properties.

## Results

### Biomimetic transfer of the starfish skeleton principles

**Fig. 1:**
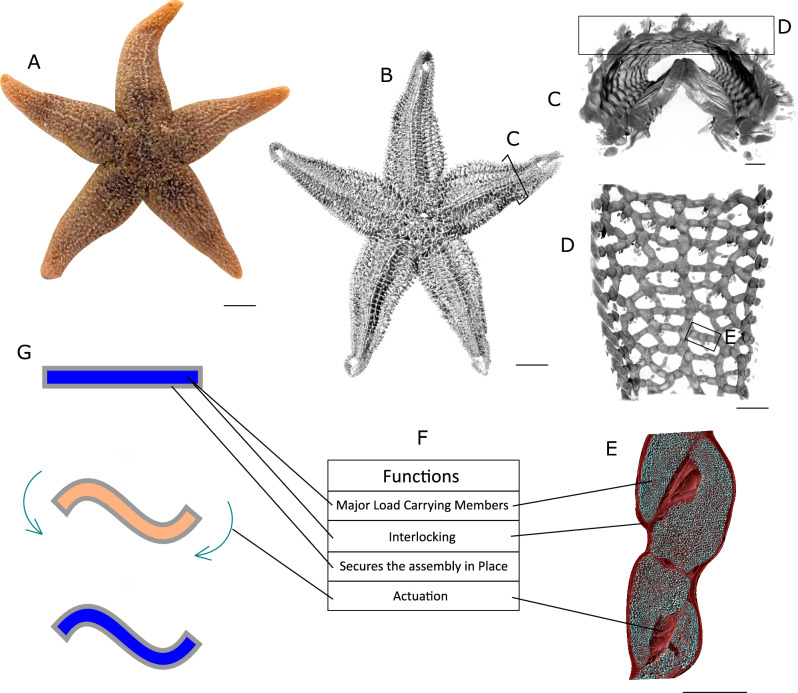
(**A**) Photograph showing the body shape of an adult *Asterias rubens*, a commonly found starfish. (**B**) X-ray CT scan of the ossicular network of the complete skeleton of the starfish *Asterias rubens*. Thousands of ossicles arrange themselves to form a mesh-shaped pattern on the aboral side. (The scan was performed at 76 µm voxel size and is adapted from^32^). (**C**) Cross-sectional view of an individual arm of the starfish showing the arrangement of the ossicles around the cavity of the arm. (13 µm voxel size, scan data adapted from^32^). (**D**) Aboral inside view of the mesh-shaped pattern showing the grid-like connection of the single ossicles. (**E**) A high-resolution X-ray CT scan of the ossicle assembly shows the inteossicular muscles and the collagenous tissues (red) surrounding each ossicle. (**F**) Table summarizing the individual functional roles of the different components. (**G**) Thermoplastic mesh below glass transition temperature (Tg) is shown in blue color and is surrounded by an elastomer jacket, shown in grey color. Below Tg, the thermoplastic mesh is rigid and can maintain its geometry. The thermoplastic mesh above Tg is shown in orange color. Above Tg, the rigidity of the thermoplastic mesh drops and the structure can be brought to a new posture by the application of mechanical forces. The structure is held in the new posture until the temperature drops down below Tg. Once the temperature is below Tg, the structure will hold its new posture without requiring any additional energy or control. Scale bars: (**A**,**B**) 10 mm; (**C**,**D**) 1 mm; (**E**) 200 µm.

The starfish skeleton is a complex biological system with distinct tissues contributing to flexibility and posture-holding. The rigid ossicles offer structural support and are the primary load-bearing elements, sharing contact points and transferring load throughout the skeleton (Fig. 1E). Inter-ossicular muscles (IOMs), connecting adjacent ossicles, act as actuators to facilitate movement and shape change (Fig. 1E). When IOMs contract, they reposition the ossicles, resulting in a shape transformation of the skeleton. This network of ossicles and IOMs is connected and surrounded by mutable collagenous tissues (MCTs), which are fibrous in structure (Fig. 1E). Under normal conditions, MCTs allow ossicle movement, permitting the starfish to change its shape. However, when the MCT fibers undergo cross-linking, they form a jammed network that locks the entire skeleton in that specific configuration. This energy-efficient posture-holding mechanism is a key feature we sought to mimic in our biomimetic design. Finally, the starfish's derma encapsulates the entire skeletal system, providing both the flexibility needed for shape changes and the cohesiveness to maintain structural integrity, while offering protection from the external environment.

Inspired by this natural design, we developed a morphing structure with two core components, a thermoplastic and an elastomer (Fig. 1G). The thermoplastic serves a dual role, mimicking both the load-bearing ossicles and the posture-locking MCTs. Below the glass transition temperature (Tg), it provides rigidity, effectively locking the morphing structure into a specific shape. Above Tg, it softens, enabling shape change. Being the major load-bearing member, its material properties (such as Young's modulus) and structural geometry, are the primary factors determining the structural stiffness of the morphing structure. Additionally, the thermoplastic's ability to melt and re-solidify enables the potential for self-healing within the structure in cases of cracks or fractures. The elastomer jacket, mimicking the starfish's derma, encapsulates the thermoplastic. Encapsulation protects the thermoplastic from the external environment and avoids its leakage during the healing process, when the thermoplastic is in its molten state. Its flexibility is crucial for enabling shape changes, while its cohesiveness maintains the structural integrity of the composite morphing structure. Additionally, the elastomer's properties contribute to the shape memory capability of the overall morphing structure. Table 1 summarizes the biomimetic design translation, mapping the functional roles of starfish skeletal elements to their respective technical components in the morphing structure.

**Table 1:** Summary of the biomimetic design concept of a starfish-inspired morphing structure. The functional principles of the starfish skeleton are mapped to corresponding technical components.

Starfish skeletal element	Function	Technical component
Ossicles	Major load-bearing	Thermoplastic mesh (below Tg)
Inter-ossicular muscles (IOMs)	Actuation for shape change	External force
Mutable collagenous tissues (MCTs)	Stiffness control for posture locking and flexibility	Thermoplastic mesh
Derma	Encapsulation, protection	Elastomer jacket

### Mechanical and thermal characterization

**Fig. 2:**
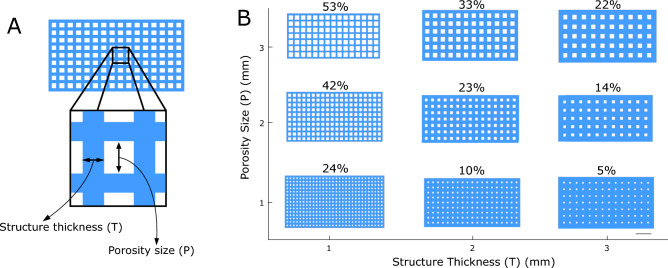
Design variations of the thermoplastic mesh. (**A**) Design variables used to manufacture prototypes were porosity size (P) and structure thickness (T). (**B**) CAD generated images of nine meshes with varying combinations of T and P values (1 mm to 3 mm). The values on the top of the meshes indicate their porosity percentage. Scale bar: (**B**) 10 mm.

The internal thermoplastic mesh of our starfish-inspired morphing structure plays a key role in determining its overall mechanical properties. To experimentally characterize the effect of the mesh design, nine unique mesh variations were designed by systematically altering the porosity size (P) and structure thickness (T) (see Fig. 2).

**Fig. 3:**
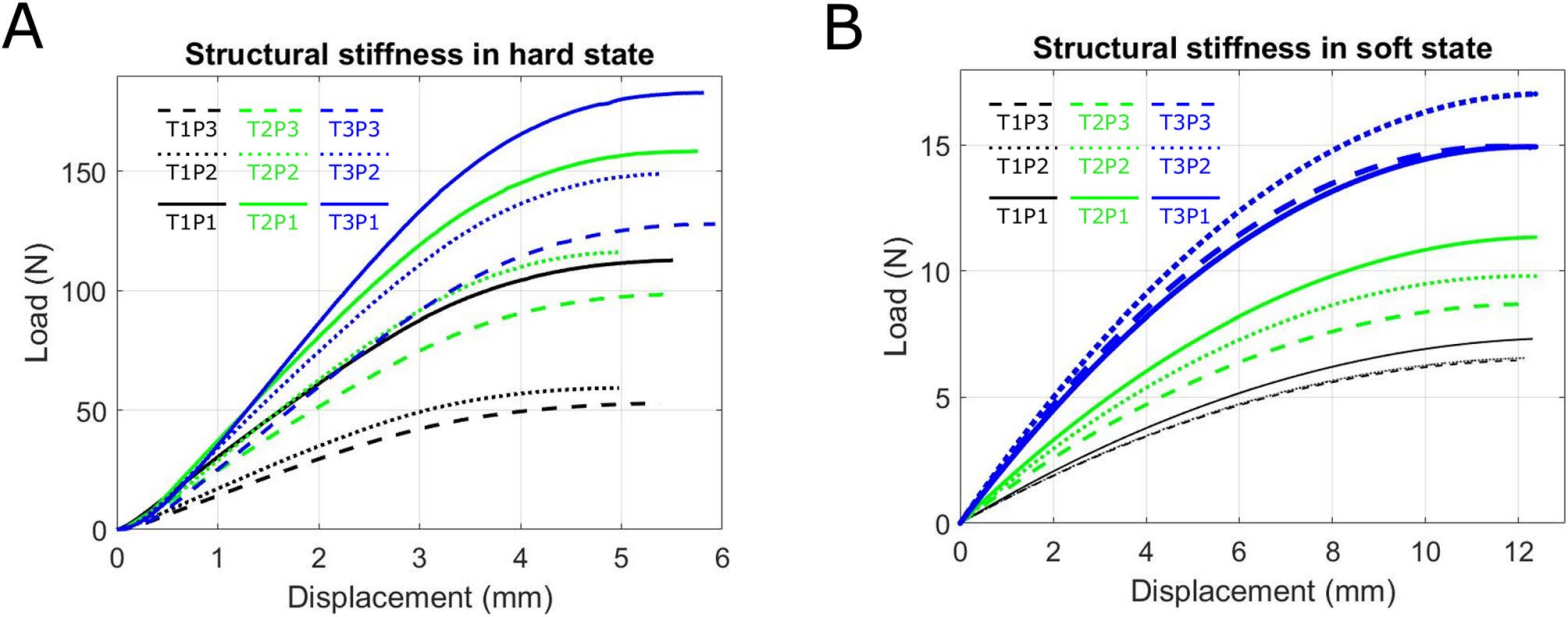
Mechanical characterization of thermoplastic meshes and prototypes (**A**) Experimentally measured load-versus-displacement curves for the thermoplastic meshes at room temperature under 3-point bending. Within the tested region all mesh combinations showed a mostly linear relation of displacement and load. (**B**) When the samples were submerged in water around the glass transition point of the PLA mesh (68 ± 1 °C) for five minutes, the structural stiffness of all prototypes was reduced by about 90%.

To experimentally characterize the effect of temperature on the structural stiffness of the prototypes, we performed 3-point-bend test (Supplementary Figure 1). At room temperature, all tested mesh structures show a mostly linear load–displacement relation (see Fig. 3A). When the prototypes were submerged in a water bath close to the glass transition point of PLA the structural stiffness of all tested prototypes decreased by about 90% (68 ± 1 °C for five minutes, see Fig. 3B and Supplementary Figure 1).

### Demonstration of time-dependent morphing ability and self-healing

To illustrate morphing capability of our starfish-inspired prototypes we characterized both the ability to retain a given shape and the ability to recover to the initial shape.

**Fig. 4:**
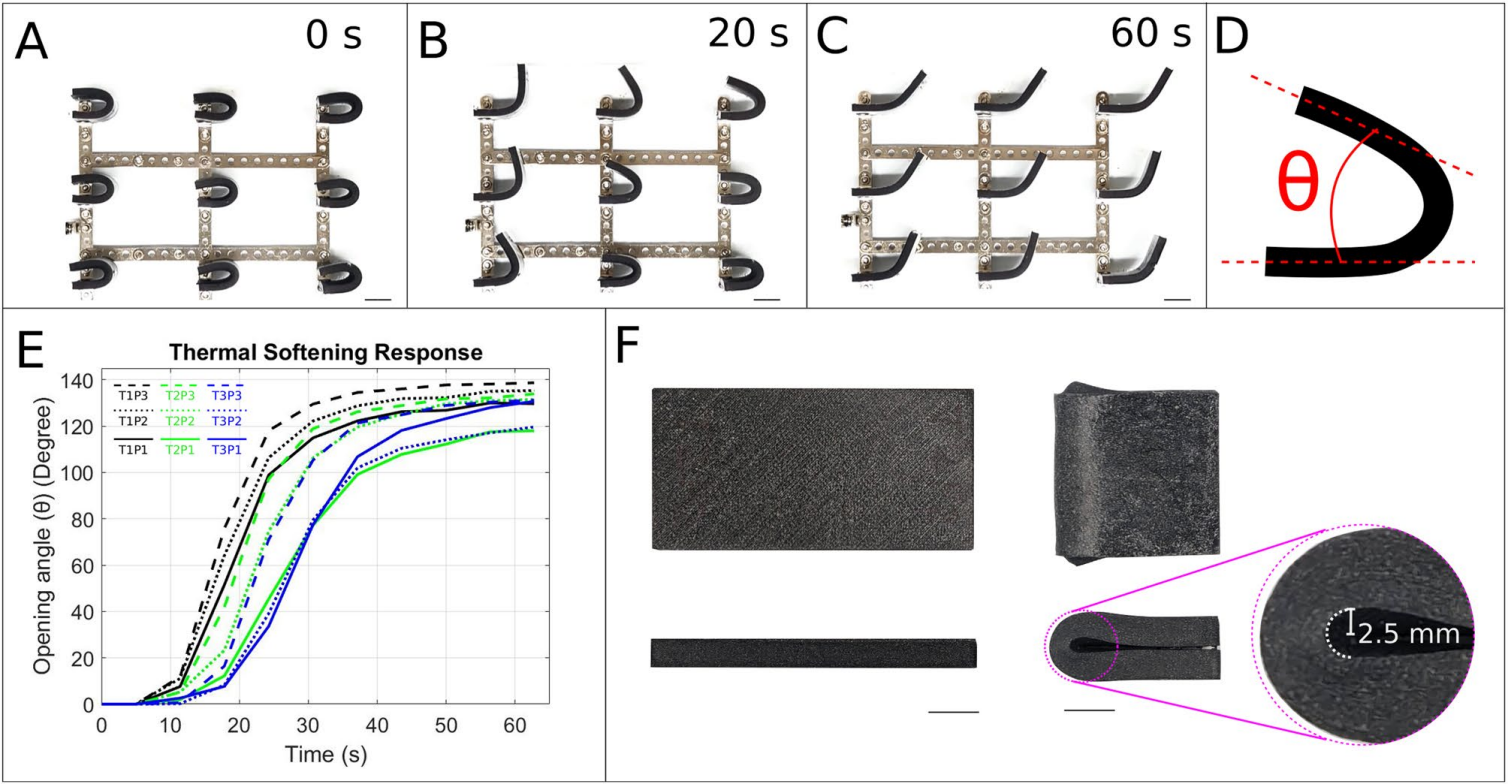
Illustration of the 4D shape memory effect of the starfish-inspired morphing structures. (**A**) Several identical prototypes with different internal mesh structures shapes were manually deformed to a bending radius of 10 mm, cooled down to room temperature, fixed at their base and then again placed in a water bath at 78 °C. (**B**) When the stiffness of the PLA mesh decreased, the prototypes started to re-take their initial shape. However, the speed of the process depends on the internal mesh structure. After 20 s the time-delay is already clearly visible. See supplemental video 1. (**C**) The shape at the end of 60 s, where the PLA meshes in all prototypes has softened. (**D**) Opening angle (θ) characterises the shape of the morphing structure at any point in time. It is the angle between the fixed half and the moving half of the structure. (**E**) The tracked opening angle (θ) of morphing structures over time. (**F**) Front and side views of the T2P2 morphing structure before and 30 min after shape transformation. The inset highlights the minimum bending radius (2.5 mm) achieved, demonstrating the structure's shape retention capability. The T2P2 design was chosen due to its average porosity and structure thickness, providing a good balance between flexibility for shape change and stiffness for shape retention. Scale bar: (**A**–**C**) 20 mm; (**F**) 10 mm.

Thermoplastic meshes were 3D-printed in a flat configuration. To demonstrate the ability to hold a given form a softened prototype was then folded in half, inducing a bending radius of nearly zero mm and thus maximum of bending stress within the mesh (Fig. 4F). After 30 min at room temperature, the bending radius was still only 2.5 mm, clearly demonstrating the prototype’s ability to maintain even extreme deformations shape without external support.

To demonstrate the time-dependent ability of our morphing structures to passively regain their initial shape, the initially flat prototypes were deformed into extreme geometries. After cooling down to room temperature the recovery response was triggered by submerging the folded structures in a hot water bath. Our results show that prototypes with higher percentage porosities exhibited faster recovery, while lower percentage porosity structures recovered more slowly (see Fig. 4 and supplemental video 1). Within 60 s, a maximum opening angle of 140° was achieved, demonstrating almost full passive shape recovery.

**Fig. 5:**
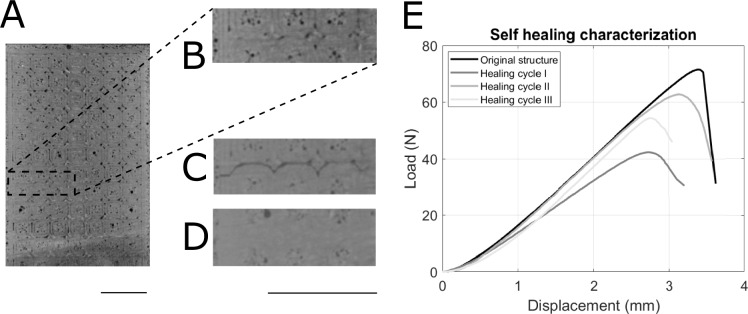
Self-healing characterization of a starfish-inspired morphing structure. (**A**) X-ray CT image of the T2P2 structure before fracture, showing its intact form and the internal 3D-printed pattern in the PLA. (**B**) Enlarged view of the T2P2 structure before fracture. (**C**) X-ray CT image of the structure after the 3-point-bend fracture test, visually confirming the presence of a crack. (**D**) X-ray CT image after the re-heating of the PLA mesh, demonstrating crack closure and the disappearance of the 3D-printed pattern due to layer fusion. (**E**) Load–displacement curves from 3-point-bend tests comparing the structure in its original state with its state after three heating cycles. Following healing, the stiffness of the structure ranged from 73 to 94% of the original, the fracture load varied from 59 to 76%, and the deflection before fracture ranged from 81 to 92% of the original structure. Scale bars: (**A**–**E**) 10 mm.

To demonstrate the ability of our prototypes to repair defects in the thermoplastic mesh structure (self-healing) we induced a controlled fracture within the PLA mesh using a 3-point-bend test. X-ray scans and the load-versus-displacement curve obtained during this test confirmed the structural failure (see Fig. 5). The fractured structure was then heated up to 200 °C for 20 min. This temperature surpasses the melting point of the thermoplastic (163 °C), allowing the polymer chains to regain mobility and potentially repair the fractured bonds. We conducted a series of three healing cycles on the prototype. After each cycle, the self-healing effectiveness was evaluated. The X-ray CT scan presented in (Fig. 5D) illustrates the structure after the first healing cycle, confirming successful crack closure. Subsequent fracture tests after each healing cycle demonstrated the recovery of structural integrity, as evidenced by the load–displacement curves shown in Fig. 5E.

## Discussion

This study presents a novel bio-inspired morphing structure inspired by the self-locking ability of the starfish skeleton. The morphing structure design employs only two easily fabricated components: a thermoplastic mesh and an elastomeric jacket. The thermoplastic mimics both the load-bearing ossicles and the posture-locking mutable collagenous tissues of the starfish skeleton. The elastomer, analogous to the starfish's derma, provides encapsulation, and protection from the external environment and enables controlled shape change (Fig. 1).

By systematically varying the geometric design of the thermoplastic mesh, we achieved precise control over structural stiffness, time-dependent thermal softening response, and ultimately, morphing behavior (Figs. 2, 3, and 4). This level of control over morphing behavior distinguishes our work from previous research, where 4D effects were typically induced through direct 3D printing of thermoplastic materials, either alone or in combination with geometric design^15,16,33^. Additionally, some studies have utilized hinges made from elastomer materials that store energy by accumulating strain or deformation during the folding process. When the external stimulus is removed or altered, the stored energy in these hinges is released, causing the structure to revert to its original shape or maintain a new configuration, depending on the design^34^. In our case, the time-dependent shape memory behavior of our prototypes stems from the interplay between the thermoplastic mesh geometry and the silicone rubber jacket. This allows us to precisely control morphing behavior by tailoring the mesh geometry. Meshes with greater porosity relative to structure thickness exhibited lower stiffness and faster thermal response, ideal for rapid shape change. Conversely, designs with thicker structures relative to porosity offered higher stiffness and slower thermal response, suitable for applications requiring robust shape retention. Interestingly, within the group of structures with comparable porosity and structure thickness (T1P1, T2P2, and T3P3), all displayed similar stiffness. However, the mesh with the finest structure (T1P1) exhibited a faster thermal response compared to T2P2 and T3P3 (Figs. 3 and 4). This could be attributed to the enhanced heat transfer through the finer mesh, allowing the thermoplastic to reach a higher temperature more quickly and thus soften more rapidly. Across all designs, we achieved a significant stiffness reduction of over 92% upon transitioning from the hard to soft state, highlighting the structure's adaptability (Fig. 3).

In addition, our morphing structure demonstrates noticeable self-healing properties. X-ray CT scans confirmed complete crack closure following thermal treatment (Fig. 5A–D). Although there are some variations in stiffness and load to failure in the healed structure during different healing cycles (Fig. 5E), these can likely be attributed to the general behavior of the thermoplastic during the re-heating process. As the 3D-printed layers melt and fuse, slight geometric changes occur in the healed structure compared to the original. Furthermore, the reduction in the mechanical properties may be due to thermoplastic degradation from repeated thermal cycling^35–38^. Despite these slight variations, the self-healing capability of our structure substantially enhances its reusability and overall lifespan.

Our morphing structure balances design simplicity with exceptional functionality. It demonstrates self-locking, continuous bending with a low radius of curvature, scalable and customizable design, self-healing, and time-dependent shape memory effects. These properties, combined with its ease of fabrication and low production costs, make it a highly promising candidate for diverse applications in fields like robotics, biomedical devices, prosthetics, aerospace, and beyond. To demonstrate the versatility of our morphing structure, we created a hand-shaped prototype. This design showcased our ability to tailor both the mechanical properties and shape memory behavior of individual "fingers”, enabling complex and time-dependent programmed 4D actuation (see supplemental video 2).

Our current prototypes open up several avenues for future research. Expanding the range of stimuli beyond heat to include light, electricity, or other triggers could enable novel actuation methods. Additionally, exploring alternative thermoplastic-elastomer combinations could yield tailored properties for specific applications. Integrating embedded actuators directly into the morphing structure would increase control complexity. Furthermore, developing advanced control strategies could enable precise shape changes and complex morphing sequences. Finally, investigating the tessellation of morphing structure units could lead to the design of large-scale morphing structures for countless applications.

## Methods

### X-ray CT scans

#### Sample preparation

Samples of the starfish *Asterias rubens* were provided by the Alfred Wegener Institute in Helgoland, where they are caught as bycatch in scientific studies. Adhering to established procedures, the starfish samples were first relaxed in freshwater and then preserved in a solution containing 4% formaldehyde and seawater sourced from the North Sea. After fixation, the specimens were permanently stored in a solution of 4% formaldehyde and distilled water. Subsequently, they were transported to the University of Applied Sciences in Bremen, Germany. Upon arrival, ossicles were carefully extracted from the aboral regions of the starfish's body wall. These ossicles were then carefully placed in a small plastic container, stabilized and supported with tissue, and scanned in an air environment.

#### Scanning

The overview scan of the complete starfish skeleton (Fig. 1B) was performed at 76 µm voxel size with a Phoenix micro-CT scanner (v|tome|x m research edition, GE Digital Solutions, 130 kV and 100 µA, 2024 projections, 0.33 s exposure time). The associated reconstruction software (Phoenix dato|× 2, GE Digital Solutions, Boston, USA) was used for the post-processing of the scans. The scan data used in this study was re-analyzed from Schwertmann et al.^32^.

Selected regions of the starfish ray (Fig. 1C,D) were scanned at 13 µm voxel size with ZEISS Xradia Versa 520 XRM machine (70 kV and 86 µA, 3201 projections, 1.5 s exposure time and 4× optical magnification). ZEISS Reconstructer Scout-and-scan software was used for reconstruction. This specific scan data was re-used from Schwertmann et al.^32^.

The high-resolution X-ray CT scan of the ossicle assembly (Fig. 1E) was performed at 1 µm voxel size with ZEISS Xradia Versa 520 XRM machine (80 kV and 88 µA, 3000 projections, 4× optical magnification). For these scans, eleven seconds of exposure time per projection was used for high contrast. ZEISS Reconstructer Scout-and-scan software was used for reconstruction. Table 2 summarizes the scan parameters for all the different scans.

**Table 2:** Summary of scan parameters for different X-ray CT scans used in this study. X-ray parameters used showed the best possible contrast of the starfish ossicles and the surrounding tissue

Scan	Voxel size (µm)	Scanner	X-rays (kV, µA)	Projections	Exposure time (s)	Reconstruction software
Overview scan—complete starfish	76	Phoenix micro-CT scanner v|tome|x m	130, 100	2024	0.33	Phoenix dato|× 2
High-resolution scan—starfish ray	13	ZEISS Xradia Versa 520 XRM	70, 86	3201	1.5	ZEISS Reconstructer Scout-and-scan
High-resolution scan—ossicle assembly	1	ZEISS Xradia Versa 520 XRM	80, 88	3000	11	ZEISS Reconstructer Scout-and-scan

### Material selection and design process

For the manufacturing process, we focused on readily available and established materials and methods wherever possible. PLA is the most commonly available and used 3D printed material, that offers excellent 3D printability^39^. Therefore, for the 3D printed mesh we used a commercially available Polylactic acid (PLA) filament (PolyTerra Savannah Yellow) (Prusa i3 MK3S FDM printer, 100% infill, 0.15 mm layer height, T_g_ 60.6 °C). For the surrounding jacket, smooth-On Dragon Skin 30 silicone resin was selected for its low viscosity and long pot life (45 min), beneficial for infiltrating the finer porosities of the thermoplastic mesh. To enhance thermal conductivity, highly conductive graphite powder (ProGraphiteShop, Article No: PG1601) was added to the resin.

### Mechanical characterization

A TA Electroforce LM1 TestBench was used to perform the mechanical testing. A 3-point bending test was performed on samples with 30 × 60 mm mesh size to evaluate the stiffness of the thermoplastic mesh and the morphing structures at room temperature and in hot water (see Supplementary Figure 1). To determine structural stiffness for both thermoplastic meshes and morphing structures, the linear range (0 to 2.5 mm displacement at 1 mm s^−1^) of the load-versus-displacement test data was analyzed (Fig. 3). Due to constraints in completing the mesh patterns, slight variations in thermoplastic mesh structure widths were unavoidable. To account for these variations, a small correction factor ((30 mm)/(Actual Width of Structure)) was introduced and multiplied to experimentally obtained load data from 3-point bend tests.

To observe the shape recovery response, folded morphing structures were first secured to a custom metallic frame, with one-half fixed in place. The frame with attached structures was then submerged in a hot water bath (78 °C) to trigger thermal softening (see Fig. 4A–C). The shape recovery process was recorded from above using a camera (supplemental video 1).

## Supplementary Information

Supplementary Figure 1.

Supplementary Video 1.

Supplementary Video 2.

## Data Availability

The datasets used and analyzed during the current study are available from the corresponding author on reasonable request.

## References

[1] Kuder, I. K., Arrieta, A. F., Raither, W. E. & Ermanni, P. Variable stiffness material and structural concepts for morphing applications. *Prog. Aerosp. Sci.* **63**, 33–55. 10.1016/j.paerosci.2013.07.001 (2013).

[2] Sofla, A. Y. N., Meguid, S. A., Tan, K. T. & Yeo, W. K. Shape morphing of aircraft wing: Status and challenges. *Mater. Des.* **31**, 1284–1292. 10.1016/j.matdes.2009.09.011 (2010).

[3] Daynes, S. & Weaver, P. M. Review of shape-morphing automobile structures: Concepts and outlook. *Proc. Inst. Mech. Eng. Part D J. Automob. Eng.* **227**, 1603–1622. 10.1177/0954407013496557 (2013).

[4] Chen, F. et al. The study on the morphing composite propeller for marine vehicle. Part I: Design and numerical analysis. *Compos. Struct.* **168**, 746–757. 10.1016/j.compstruct.2017.02.072 (2017).

[5] Barlas, A. & Madsen, H. Atmospheric full scale testing of a morphing trailing edge flap system for wind turbine blades. In *In Proceedings. 26th International Conference on Adaptive Structures and Technologies* (APA, 2015).

[6] Baier, H. High precision adaptive space structures. *Adv. Sci. Technol.* **83**, 115–121. 10.4028/www.scientific.net/ast.83.115 (2012).

[7] Barbarino, S., Bilgen, O., Ajaj, R. M., Friswell, M. I. & Inman, D. J. A review of morphing aircraft. *J. Intell. Mater. Syst. Struct.* **22**, 823–877. 10.1177/1045389X11414084 (2011).

[8] Zhang, X. & Zhu, B. Introduction to compliant mechanisms and design methods. In *Topology Optimization of Compliant Mechanisms. Springer, Singapore.*10.1007/978-981-13-0432-3_1 (2018).

[9] Howell, L. L. Compliant mechanisms. In *21st Century Kinematics* (ed. McCarthy, J.) 189–216 (Springer London, 2013). 10.1007/978-1-4471-4510-3_7.

[10] Sydney Gladman, A., Matsumoto, E. A., Nuzzo, R. G., Mahadevan, L. & Lewis, J. A. Biomimetic 4D printing. *Nat. Mater* **15**, 413–418. 10.1038/nmat4544 (2016). 26808461 10.1038/nmat4544

[11] Teoh, J. E. M. et al. Hierarchically self-morphing structure through 4D printing. *Virtual Phys. Prototyp.* **12**, 61–68. 10.1080/17452759.2016.1272174 (2017).

[12] Zhang, J. et al. Voxelated three-dimensional miniature magnetic soft machines via multimaterial heterogeneous assembly. *Sci. Robot.* **6**. 10.1126/scirobotics.abf0112 (2021). 10.1126/scirobotics.abf0112 PMC7612277 34043568

[13] Merces, L. et al. Bio-inspired dynamically morphing microelectronics toward high-density energy applications and intelligent biomedical implants. *Adv. Mater.* **36**. 10.1002/adma.202313327 (2024). 38402420 10.1002/adma.202313327

[14] Ni, X. et al. Soft shape-programmable surfaces by fast electromagnetic actuation of liquid metal networks. *Nat. Commun.* **13**, 5576. 10.1038/s41467-022-31092-y (2022). 36151092 10.1038/s41467-022-31092-y PMC9508113

[15] Bodaghi, M., Damanpack, A. R. & Liao, W. H. Adaptive metamaterials by functionally graded 4D printing. *Mater. Des.* **135**, 26–36. 10.1016/j.matdes.2017.08.069 (2017).

[16] Alshebly, Y. S. et al. Bioinspired pattern-driven single-material 4D Printing for self-morphing actuators. *Sustainability* **14**. 10.3390/su141610141 (2022).

[17] Plooij, M., Mathijssen, G., Cherelle, P., Lefeber, D. & Vanderborght, B. Lock your robot: A review of locking devices in robotics. *IEEE Robot. Autom. Mag.* **22**, 106–117. 10.1109/MRA.2014.2381368 (2015).

[18] Zhao, Y., Hao, G., Chai, L., Tian, Y. & Xi, F. A compliant-mechanism-based lockable prismatic joint for high-load morphing structures. *Mech. Mach. Theory* **178**. 10.1016/j.mechmachtheory.2022.105083 (2022).

[19] Lu, S. et al. Design and testing of a highly reconfigurable fixture with lockable robotic arms. *J. Mech. Des.* **138**(8). 10.1115/1.4033037 (2016).

[20] Li, Y. & Pellegrino, S. A theory for the design of multi-stable morphing structures. *J. Mech. Phys. Solids* **136**. 10.1016/j.jmps.2019.103772 (2020).33518805

[21] Zhang, Z. et al. Bistable morphing composite structures: A review. *Thin-Walled Structures* **142**, 74–97. 10.1016/j.tws.2019.04.040 (2019).

[22] Li, S. & Wang, K. W. Plant-inspired adaptive structures and materials for morphing and actuation: A review. *Bioinspir. Biomim.* **12**(1). 10.1088/1748-3190/12/1/011001 (2017). 27995902 10.1088/1748-3190/12/1/011001

[23] Siéfert, E., Reyssat, E., Bico, J. & Roman, B. Bio-inspired pneumatic shape-morphing elastomers. *Nat. Mater.* **18**, 24–28. 10.1038/s41563-018-0219-x (2019). 30455447 10.1038/s41563-018-0219-x

[24] Kim, W. et al. Bioinspired dual-morphing stretchable origami. *Sci. Robot.* **4**. 10.1126/scirobotics.aay3493 (2019). 33137780 10.1126/scirobotics.aay3493

[25] O’neill, P. Structure and mechanics of starfish body wall. *J. Exp. Biol.* **147**(1), 53–89. 10.1242/jeb.147.1.53 (1989). 2614339 10.1242/jeb.147.1.53

[26] Ji, C., Wu, L., Zhao, W., Wang, S. & Lv, J. Echinoderms have bilateral tendencies. *PLoS ONE* **7**. 10.1371/journal.pone.0028978 (2012). 22247765 10.1371/journal.pone.0028978 PMC3256158

[27] Wilkie, I. C. Mutable collagenous tissue: Overview and biotechnological perspective. *Prog. Mol. Subcell. Biol.* **39**, 221–250. 10.1007/3-540-27683-1_10 (2005). 17152700 10.1007/3-540-27683-1_10

[28] Mo, J. et al. Interfibrillar stiffening of echinoderm mutable collagenous tissue demonstrated at the nanoscale. *Proc. Natl. Acad. Sci.* **113**(42), E6362–E6371. 10.1073/pnas.1609341113 (2016). 27708167 10.1073/pnas.1609341113 PMC5081653

[29] Gayathri, S. et al. In vitro study of magnesium-calcite biomineralization in the skeletal materials of the seastar *Pisaster giganteus*. *Chemistry* **13**(11), 3262–3268. 10.1002/chem.200600825 (2007). 17205593 10.1002/chem.200600825

[30] Weber, J. N. The incorporation of magnesium into the skeletal calcites of echinoderms. *Am. J. Sci.* **267**(5), 537–566. 10.2475/ajs.267.5.537 (1969).

[31] Blowes, L. M. et al. Body wall structure in the starfish *Asterias rubens*. *J. Anat.* **231**(3), 325–341. 10.1111/joa.12646 (2017). 28714118 10.1111/joa.12646 PMC5554833

[32] Schwertmann, L., Focke, O. & Dirks, J. H. Morphology, shape variation and movement of skeletal elements in starfish (*Asterias rubens*). *J. Anat.* **234**, 656–667. 10.1111/joa.12964 (2019). 30861581 10.1111/joa.12964 PMC6481417

[33] Bodaghi, M., Noroozi, R., Zolfagharian, A., Fotouhi, M. & Norouzi, S. 4D printing self-morphing structures. *Materials* **12**(8). 10.3390/ma12081353 (2019). 31027212 10.3390/ma12081353 PMC6515691

[34] Zhang, W. et al. Unlocking micro-origami energy storage. *ACS Appl. Energy Mater.*10.1021/acsaem.4c00702 (2024). 39734918 10.1021/acsaem.4c00702 PMC11672231

[35] Mortazavian, S., Fatemi, A., Mellott, S. R. & Khosrovaneh, A. Effect of cycling frequency and self-heating on fatigue behavior of reinforced and unreinforced thermoplastic polymers. *Polym. Eng. Sci.* **55**, 2355–2367. 10.1002/pen.24124 (2015).

[36] Constable, I., Williams, J. & Burns, D. Fatigue and cyclic thermal softening of thermoplastics. *J. Mech. Eng. Sci.* **12**(1), 20–29. 10.1243/JMES_JOUR_1970_012_006_02 (1970).

[37] Hancox, N. L. Thermal effects on polymer matrix composites: Part 2. Thermal degradation. *Mater. Des.* **19**(3), 93–97. 10.1016/S0261-3069(98)00019-3 (1998).

[38] Hancox, N. L. Thermal effects on polymer matrix composites: Part 1. Thermal cycling. *Mater. Des* **19**(3), 85–91. 10.1016/S0261-3069(98)00018-1 (1998).

[39] Joseph, T. M. et al. 3D printing of polylactic acid: Recent advances and opportunities. *Int. J. Adv. Manuf. Technol.* **125**, 1015–1035. 10.1007/s00170-022-10795-y (2023). 36644783 10.1007/s00170-022-10795-y PMC9822698

