# Supplementary figures and images for: A starfish-inspired 4D self-healing morphing structure
Source: Sci Rep. 2024 Sep 25;14:22024. doi: 10.1038/s41598-024-71919-w (PMC11424623; doi:10.1038/s41598-024-71919-w)

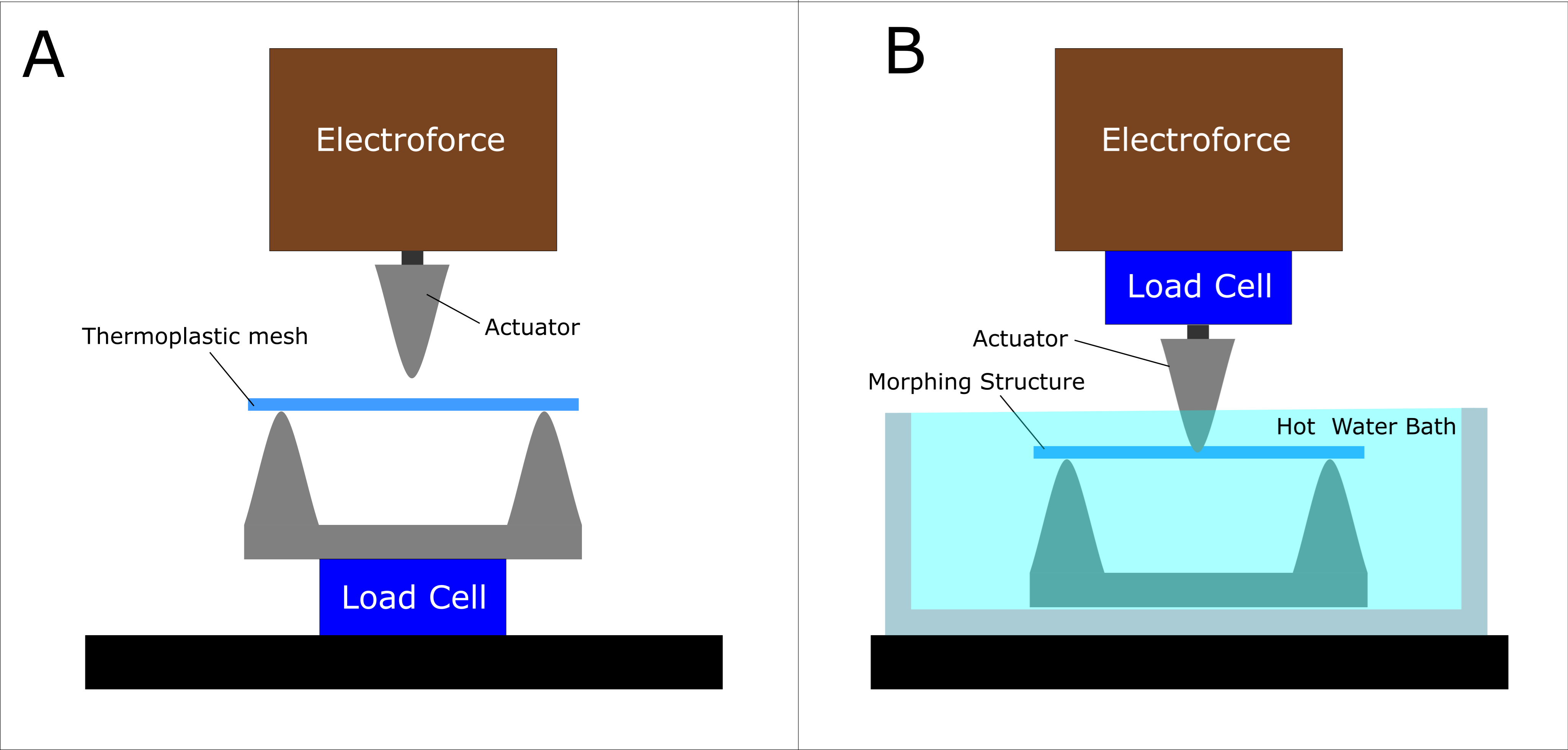

Supplement: Supplementary file 1 — Supplementary Figure 1. [file 41598_2024_71919_MOESM1_ESM.png]
